# Optimal autofluorescence blocking for reliable immunofluorescence in human ovarian samples: A comparison of 4 blocking kits

**DOI:** 10.1016/j.bbrep.2026.102762

**Published:** 2026-08-24

**Authors:** Rebekka Einenkel-Ulrich, Johanna Schlingheider, Cara Maria Färber, Andreas Schallmoser, Nicole Sänger

**Affiliations:** University of Bonn, University Hospital Bonn, Department of Gynecological Endocrinology and Reproductive Medicine, Venusberg-Campus 1, Bonn, 53127, Germany

**Keywords:** Ovary, Immunofluorescence, Quenching autofluorescence, Blocking autofluorescence, VEGF-A, IL-8

## Abstract

**Introduction:**

Fertility depends on the complex process of folliculogenesis, yet many aspects of early human follicle development remain unclear. Immunohistofluorescence (IHF) enables detailed analysis of follicular structures and allows quantification. However, it is often affected by tissue autofluorescence. This study compares four commercially available kits to identify the most effective method for reducing autofluorescence and improving IHF accuracy.

**Methods:**

Sections of human ovarian tissue were treated according to the staining protocol without antibodies, but with one of four kits reducing autofluorescence to compare their efficacy. Images were taken on the Ti2-E fluorescence microscope in three different fluorescence channels.

The kit with the highest reduction in autofluorescence was applied for a strong and a rather subtle staining in order to analyze the signal-to-background ratio (SBR).

**Results:**

All tested reagents reduced tissue autofluorescence in all three fluorescence channels. Among the evaluated products, TrueBlack showed the greatest reduction in autofluorescence. When combined with IHF staining, it significantly reduced background fluorescence and improved the SBR. Nevertheless, TrueVIEW and Ready Probes were similarly effective.

**Conclusion:**

Autofluorescence in human ovarian tissue can be effectively reduced using commercially available reagents. Among the tested products, TrueBlack showed the highest efficacy with our specific protocol and setup. Given the high demand for research and the rarity of suitable human ovarian samples, sharing these results may support more accurate and reproducible immunofluorescence analyses in future studies.

## Highlights

• Human ovarian tissue exhibits strong intrinsic autofluorescence in immunofluorescence assays.
• All 4 tested kits reduced autofluorescence across multiple fluorescence channels.
• TrueBlack achieved the strongest background reduction.
• Optimized blocking enhances reproducibility of immunofluorescence in scarce human samples.

## 1 Introduction

Fertility is of concern for our society, as it directly affects our future generations. Therefore, a deep understanding of its underlying mechanisms is vital. This includes the complex processes involved in growth and maturation of oocytes within the human ovary (folliculogenesis). By exploring these biological bases, we will more effectively address fertility-related challenges in health and disease.

Folliculogenesis is a complex process involving multiple cell types, remodeling of the structures and the growth and maturation of the oocyte. Although much valuable work has already been conducted investigating this multifaceted process, many details, especially concerning the very early folliculogenesis, still remain unknown [1].

Understanding the full process of folliculogenesis is aimed for several reasons including the development of *in vitro* activation, differentiation, growth and maturation approaches (IVA/IVD/IVG/IVM) and investigating the underlying mechanisms of ovarian defects as well as therapeutic approaches [[2], [3], [4], [5]].

The rarity of healthy ovarian tissue from fertile women for obvious and comprehensible reasons limits access to this research. The cryopreservation of human ovarian tissue is one of the few possibilities to get access to *ex vivo* samples of human ovarian tissue [6,7]. The examination of the contained follicles provides valuable information concerning the current state of each follicle. Although it serves information as puzzle piece by puzzle piece, it contributes to complete a whole picture of folliculogenesis.

Several methods can be used to assess the current state of tissue, cells or compartments. One vivid method includes histology as it represents structural features and adaptions in the tissue. Immunohistology staining allows to assess a punctual impression of the ongoing processes. Immunohistofluorescence (IHF) allows the staining of several parameters at once as well as their relative quantification [8,9]. The latter allows to analyze changes in the expression levels. Thus, IHF stands out from immunohistochemistry (IHC), which is a less accurate method in the context of quantification.

One of the main obstacles in IHF, however, is tissue autofluorescence, as it can impair analysis and signal significance [10,11]. In contrast to IHC, autofluorescent signals, which are shown by all tissues, can compromise the IHF staining causing false-positive signals. Several methods and reagents are described to reduce autofluorescence. Here, we aimed to compare four commercially available reagents to reduce autofluorescence to find the kit, which fits to our tissue and staining protocol requirements the most.

Tissue autofluorescence originates from both endogenous fluorophores and experimental procedures. Endogenous fluorophores include extracellular matrix components such as collagen and elastin, metabolic cofactors including flavins and NAD(P)H, certain cells such as red blood cells and adipocytes, as well as lipofuscin, which accumulates in long-lived and ageing cells. Consequently, the intensity and distribution of autofluorescence vary depending on tissue composition, donor characteristics such as age, and tissue-specific features. In addition, experimental factors, particularly aldehyde-based fixation, can introduce fluorescent reaction products that further increase background fluorescence. Aldehyde fixation generates Schiff bases and other cross-linked reaction products, some of which are fluorescent. Sample preparation as well as antigen retrieval may also influence the detection and apparent intensity of autofluorescent signals. These autofluorescent signals can be detected during IHF as well creating false-positive signals. A high signal-to-background ratio ensures conclusive analysis of the stainings.

Several chemical approaches have been developed to reduce tissue autofluorescence by targeting different sources of fluorescence. Sodium borohydride reduces aldehyde-derived fluorophores generated during fixation, whereas copper sulfate suppresses fixation-induced autofluorescence through copper ion-mediated quenching. In contrast, Sudan Black B primarily reduce autofluorescence by masking endogenous lipophilic fluorophores such as lipofuscin.

In addition to chemical quenching, autofluorescence can be reduced using imaging- and computational-based approaches. Background subtraction removes estimated background signals, spectral unmixing separates autofluorescence from fluorophore-specific signals based on their emission spectra, and computational subtraction uses autofluorescence reference images to digitally remove the autofluorescent component. While these approaches can improve image quality, they often require additional imaging, specialized software, or dedicated instrumentation. In this study, we aim to compare four different products for reducing autofluorescent signal in order to find the optimal product fitting to our sample, fixation and staining protocol. To evaluate the performance of the autofluorescence quenching reagents under different staining conditions, two representative markers were selected: VEGF-A as a marker with robust, high-intensity immunofluorescence and IL-8 as a marker with comparatively weak staining in human ovarian tissue.

## 2 Methods

### 2.1 Patients

Samples of 4 patients was used for this study (see Table 1). Ovarian tissue transported overnight in Custodiol (Dr. Franz Köhler Chemie GmbH) at 4 °C was used in this study. All patients gave their informed, written consent that up to 10 % of her removed ovarian tissue may be used for research (ethical vote number 007/09 from the Ethics Committee of the Medical Faculty of the University Hospital Bonn, University Bonn). The work has been carried out in accordance with applicable regulations and the Declaration of Helsinki.

**Table 1:** Patient characteristics, including age and indication for ovarian tissue collection.

	Number of patients	Mean Age at Explantation (min-max)	Indications
Fig. 1A–C, Fig. 2, Fig. 3, Fig. 4	1	25	Hodgkin Lymphoma
Fig. 1D–F	4	27,5 (20-38)	2x Mamma Carcinoma, 1x Hodgkin Lymphoma, 1x Non-Hodgkin Lymphoma

### 2.2 IHF

#### 2.2.1 Fixation and thawing of ovarian tissue

The ovarian cortical tissue punches (2 mm in diameter, 1 mm in height) were fixed in 3.7 % formalin (Carl Roth, Germany) in PBS (PAN-Biotech, Germany) overnight at 4 °C. After washing in PBS the tissue punches were incubated in 15 % sucrose (Sigma-Aldrich, USA) in PBS for 10 min. This process was repeated in 30 % sucrose in PBS for another 10 min. Afterwards, the tissue punches were placed in CryoGlue (SLEE, Germany) and frozen in liquid nitrogen. 5 μm tissue sections were produced using the MEV cryostat (SLEE, Germany) and carefully placed onto microscopic Superfrost Plus Adhesion Microscope Slides (PHC Holdings, Japan).

#### 2.2.2 Immunohistofluorescence staining (IHF)

1. After drying the tissue sections on the slides, they were hydrated in tap water for 5 s and incubated in 70 % ethanol (VWR Chemicals, USA) for 2 min.
2. **MaxBlock Autofluorescence Reducing Reagent Kit (MaxVision Biosciences, USA)** Reagent A was added to designated tissue sections for 5 min, while the remaining slides were left in ethanol.
3. Thereafter, all slides were briefly dipped in 60 % ethanol several times and incubated in distilled water for 5 min.
4. Slides were washed twice for 3 min in TBST (20 mM Tris base (Sigma-Aldrich, USA), 150 mM NaCl (Carl-Roth, Germany), 0.1 % Tween 20 (PanReacAppliChem, Germany) in *A. dest.*, pH = 7.6).
5. Afterwards, they were placed in Antigen Retrieval Buffer (10 mM Tris base, 1 mM EDTA (Sigma-Aldrich, USA), 0.05 % Tween 20 in *A. dest.*, pH = 9.0) for 20 min at 90 °C and rinsed in TBST after cooling for 10 min.
6. Next, the tissue sections were permeabilized (Perm. Buffer: 20 mM Tris base, 150 mM NaCl, 0.2 % Tween 20 in *A. dest.*, pH = 7.6) for 10 min on a rocking shaker (15 rpm) and rinsed in TBST.
7. Blocking was performed using 5 % skim milk powder (SMP; Sigma-Aldrich, USA) in TBST for 30 min in a hydrated chamber.
8. Depending on the experiment, primary antibodies were diluted in 5 % SMP in TBST. Otherwise as well as as staining controls were incubated in 5 % SMP in TBST only overnight at 4 °C in a hydrated chamber.
9. After washing three times for 5 min in TBST on a rocking shaker, either the secondary antibody diluted in 5 % SMP in TBST was added or just 5 % SMP in TBST was used and left for 2 h at 4 °C. Afterwards, the washing in TBST was repeated and slides were placed in distilled water for 5 min.
10. I) **MaxBlock Autofluorescence Reducing Reagent Kit (MaxVision Biosciences, USA)** Reagent B was added to the same section as in 2. and incubated for 5 min.
  II) **TrueBlack Lipofuscin Autofluorescence Quencher (biotium, USA)** was diluted (1:20) in 70 % ethanol and incubated at tissue sections for 1.5 min.
  III) For all three components (A,B,C) of the **Vector TrueVIEW Autofluorescence Quenching Kit (Vector Laboratories, USA)** the same volumes were used. First Medium A and B were combined (1:2) and then the same amount of Medium C was added. The solution was incubated for 4 min.
  IV) The same procedure as in III) was performed with **ReadyProbes Tissue Autofluorescence Quenching Kit (Thermo Fisher Scientific, USA)** and incubated for 5 min.
11. All slides were rinsed three times for 2 min in distilled water before mounting in DAPI-mounting medium (Carl-Roth, Germany).
12. Cover slips were sealed with transparent nail polish on its edges.
13. Slides were keep in the dark until examination, which was performed immediately after staining and within the same day using the Ti2-E microscope (Nikon, Japan) equipped with LED-DAPI-A-2360A Filter Cube (32 mm), LED-FITC-A-2745A Filter Cube (32 mm), LED-mCherry-A Filter Cube (32 mm) and Cy3-4040C Filter Cube (32 mm). Images of the same experiment were taken and exported with the same settings to ensure comparability of appearance and measurements.

### 2.3 Antibodies

Anti-VEGF-A antibody (1:100; rabbit IgG, article number: ab39250, Thermo Fisher Scientific, USA), anti-IL-8 antibody (1:500; rabbit IgG, article number: 17038-1-AP, Thermo Fisher Scientific, USA) and secondary antibody (F(ab')2-Goat anti-Rabbit IgG (H + L) Cross-Adsorbed, article number: A11070, fluorochrome: AF488 GFP, Thermo Fisher Scientific, USA) were used.

### 2.4 Analysis

Images were acquired with the Eclipse Ti2-E microscope (Nikon, Japan). The DAPI signal was used for orientation and focus adjustment. Microscope acquisition settings (exposure time, gain, and illumination intensity) were optimized for the respective microscope and are provided below (see Table 2). To ensure reliable detection and quantification of endogenous autofluorescence, comparatively high acquisition settings were intentionally used for autofluorescence imaging. Within each experiment, identical acquisition parameters were maintained for all samples to enable direct comparison between treatment groups. For the assessment of overall tissue autofluorescence, one overview image of each tissue section was acquired using a 20× objective, covering the majority of an entire section. The analysis of the ovarian cortical tissue sections was performed using NIS-Elements AR 5.30.03 64-bit. The region of interest (ROI) comprised the entire tissue section. The ROI was identified using the software's automatic detection tool. Obvious artifacts, including tissue folds, tears, and debris, were manually excluded from the analysis before fluorescence quantification. When examining the blood vessels and follicles, this was defined as ROI. In order to assess the signal-to-background ratio, a binary mask was created within the ROI. The mean signal intensity of the marked and the unmarked binary regions were measured. The signal-to-background ratio (SBR) was assessed as following: mean intensity (signal)/mean intensity (background) as shown in Fig. 3A.

**Table 2:** Microscope acquisition settings.

	Fluorochrome/Channel	
**Setting**	DAPI	GFP, mCherry, Cy3
Exposure Time	50 ms	500 ms
Gain	1.8x	1.8x
Illumination Intensity	25 %	75 %

### 2.5 Statistics

The graphical representations and the statistical analysis were generated with Prism 9 (Graphpad Software, USA). The evaluation was based on the assumption of a normal distribution. One-Way-ANOVA with Dunnett's multiple comparisons test and unpaired t-tests were used for the evaluation. P < 0.05 was considered significant.

## 3 Results

The goal of this study was to compare four different products for reducing autofluorescence in order to find the optimal product for our sample, fixation and staining protocol.

**Fig. 1:**
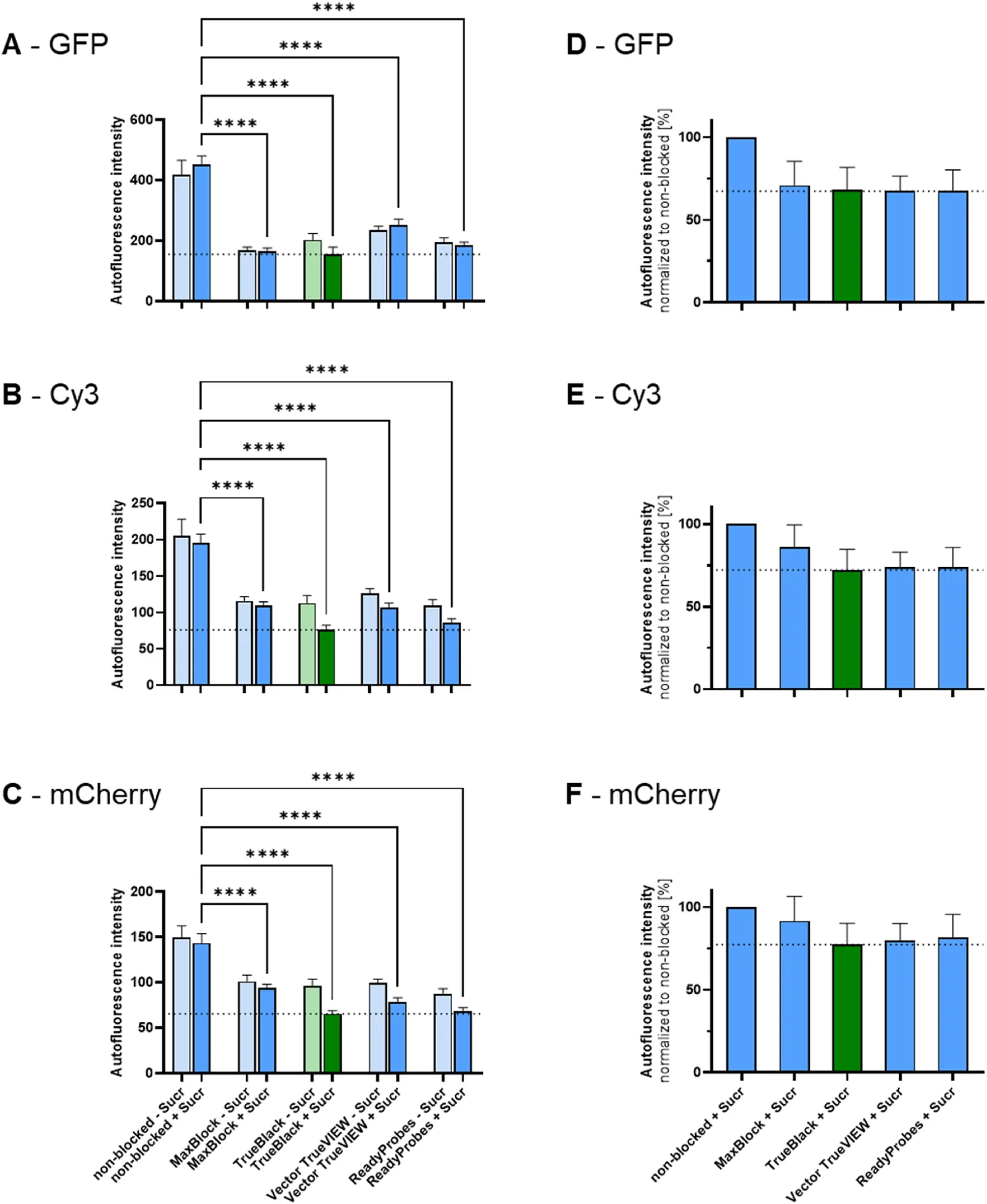
**– Reduction of autofluorescence by commercially available kits.** (**A-C**) Ovarian cortical tissue sections were prepared for immunohistofluorescence staining (IHF) and treated with or without sucrose and with the respective autofluorescence reducing kit. 10 tissue sections of one patient were evaluated using one-Way-ANOVA with Dunnett's multiple comparisons test. Each graph shows the assessed mean autofluorescence intensity of the entitled fluorescence channel: **A** – GFP, **B** – Cy3, **C**- mCherry. The dotted line marks the smallest intensity. ****p < 0.0001. Bars show Mean ± SEM. n = 10 tissue sections, with each section originating from an individual ovarian cortical tissue punch. **(D-F)** Ovarian cortical tissue was analysed either fresh or after cryopreservation and thawing (samples of 2 patients each) and processed according to our individual IHF protocol, including treatment with sucrose and with the indicated autofluorescence-reducing kit. On average, 6 tissue sections per patient and kit were analysed (range: 2-10), and the patient-specific mean was used for data presentation. Bars represent the mean autofluorescence signal normalized to the corresponding non-blocked control tissue sections ±SEM. The dotted line indicates the lowest normalized autofluorescence intensity. n = 4 patients analysed in 3 independent experiments.

To evaluate the effectiveness of the different kits, ovarian cortical tissue punches of one patient were subjected to IHF without the use of specific antibodies. The addition of all four autofluorescence reducing kits compared to the untreated control decreased the autofluorescence intensity significantly (****p < 0.0001, see Fig. 1A–C) in all three channels analysed. Especially when the sucrose incubation step was included, the autofluorescence intensity was the lowest in each channel (reduction by 65.73 %, 54.3 % and 61.01 %, respectively).

Across four patients, TrueBlack was considered the most effective autofluorescence reducing kit for our protocol requirements (see Fig. 1D–F). The autofluorescence intensity was reduced by 31.92 % in the GFP channel, 22.63 % in the mCherry channel and 29.90 % in the Cy3 channel.Overall, TrueBlack showed the highest efficacy in reducing autofluorescence in the Cy3 and mCherry channels. In the GFP channel, TrueVIEW and Ready Probes achieved a slightly greater reduction than TrueBlack (by additional 0.72 % and 0.54 %, respectively), although the overall performance of all three reagents was comparable.

**Fig. 2:**
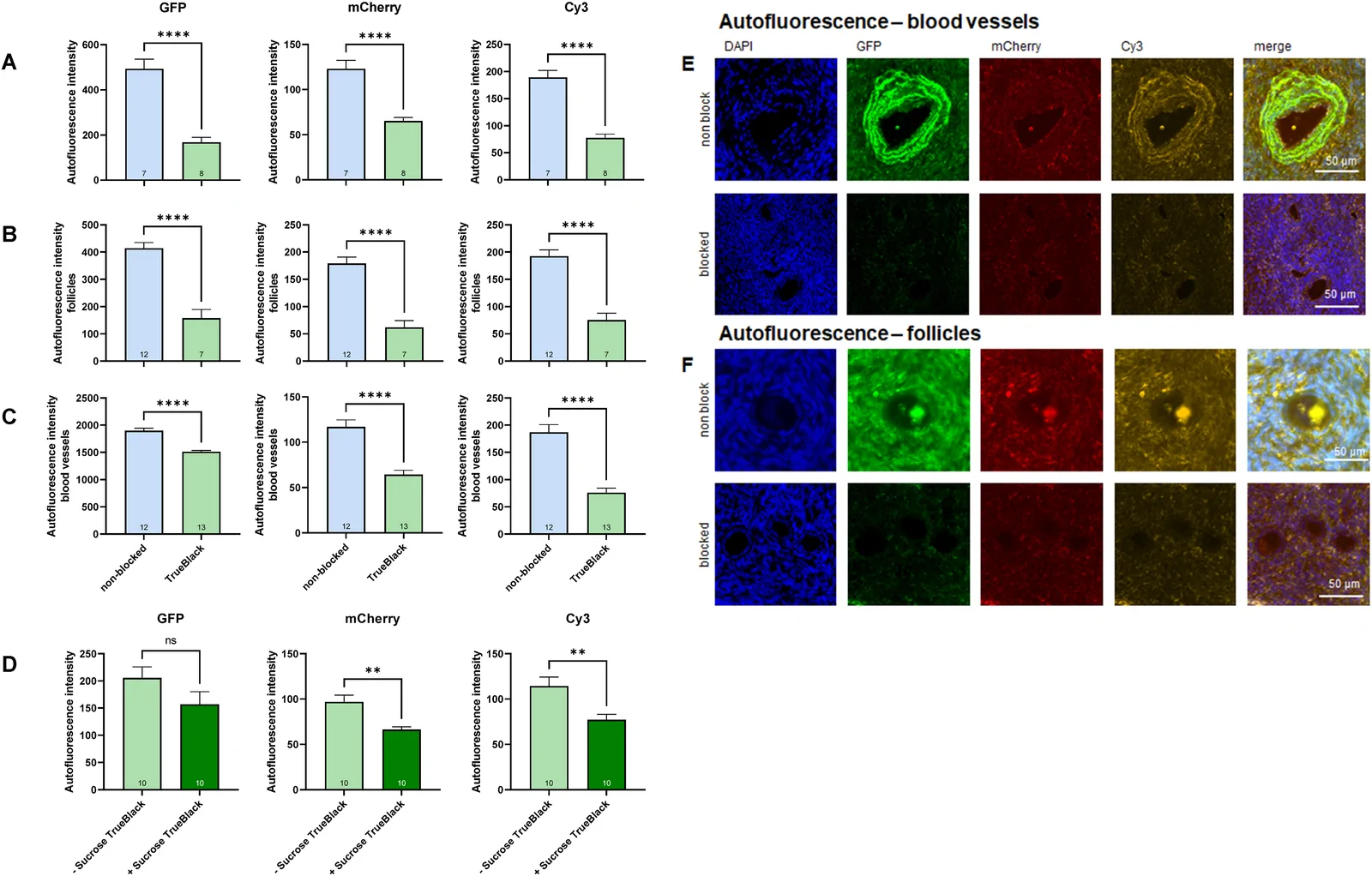
**Reduction of autofluorescence by TrueBlack with regard to vessels and follicles.** Ovarian tissue section were treated like the IHF protocol without antibody staining with or without the Autofluorescence Reducing Kit TrueBlack (Biotium). DAPI staining was conducted. Special focus is put on ovarian blood vessels (B) and follicles (C) and the application of sucrose after tissue fixation (D). Bars show Mean + SD. The number of evaluated tissue punches is shown at the bottom of the bar. Unpaired t-tests were used for the evaluation. ****p < 0.0001,**p < 0.01. Each graph shows the assessed autofluorescence intensity of the entitled fluorescence channel and structure. ****p < 0.0001. Bars show Mean ± SEM. The numbers in the bars indicate the sample size (n). For (A) and (D), n represents the number of tissue sections, with each section originating from an individual ovarian cortical tissue punch. For (B) and (C), n represents the number of follicles or blood vessels analysed within the tissue sections shown in (A). Representative images of blood vessels (E) and primordial follicles (F) in ovarian tissue are presented. All images were taken and exported with the same settings to ensure comparability. Scale bar marks 50 μm.

Subsequently, the focus was put on the effect on specific details in ovarian tissue like follicles and blood vessels. The evaluation of the blood vessels and follicle structures on multiple tissue slices of a single patient also showed that the autofluorescence intensity was significantly reduced when using TrueBlack (****p < 0.0001, see Fig. 2). The use of sucrose subsequently to the fixation process turned out to make a significant difference in reducing the autofluorescence intensity (**p < 0.01 see Fig. 2 D).

**Fig. 3:**
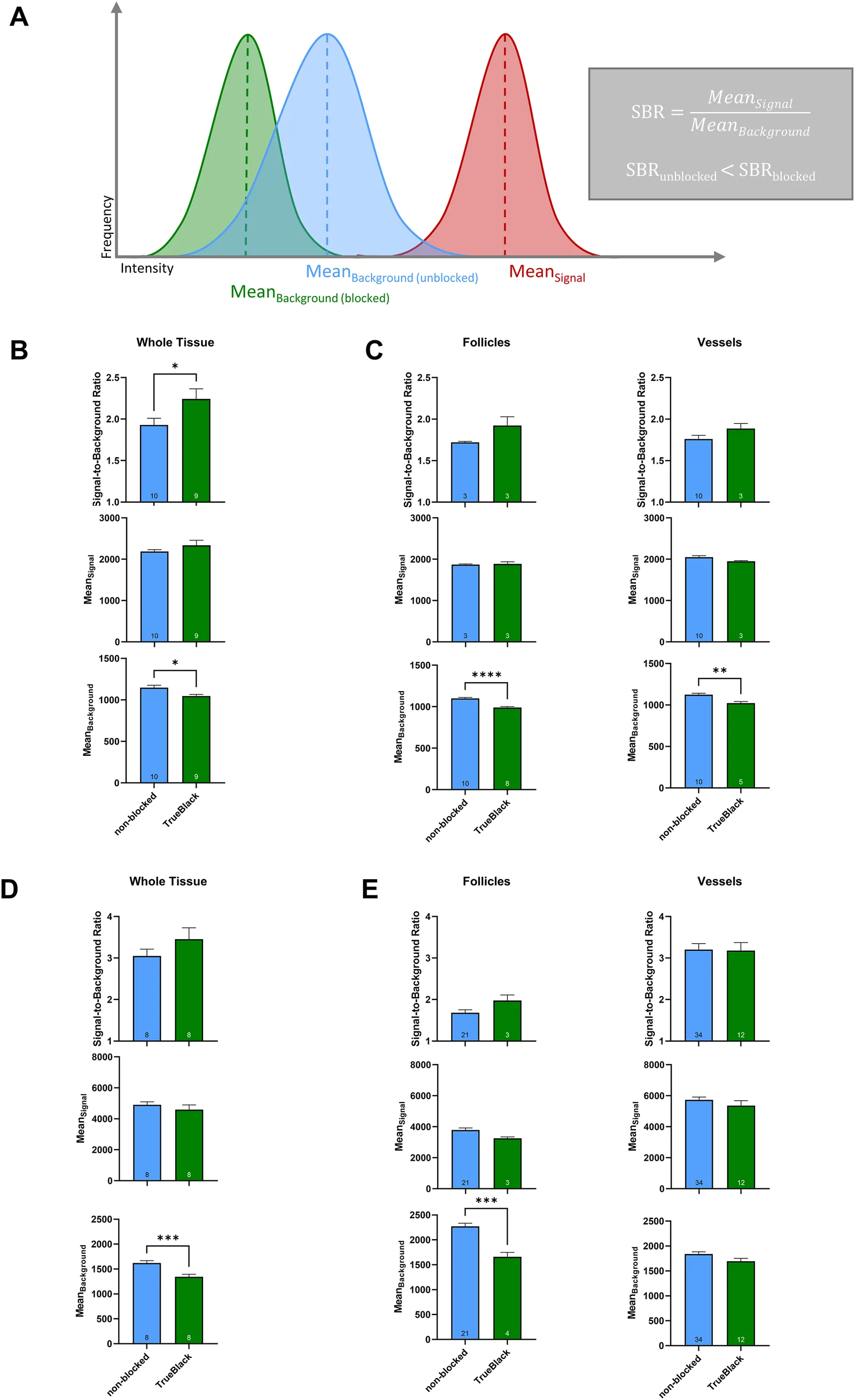
**Increased Signal-to-Background Ratio (SBR) due to reduced background staining.** (**A**) The reduction of the intensity of the background increases SBR. Immunohistofluorescence staining of IL-8 (**B-C**) and vascular endothelial growth factor (VEGF; **D-E**) in ovarian cortex punches were compared with or without autofluorescence blockage. Sections of whole tissue were assessed as well as blood vessels and follicles. Bars show Mean ± SEM. The number of evaluated tissue sections is shown at the bottom of the bar, with each section originating from an individual ovarian cortical tissue punch. Unpaired t-tests were used for statistical assessment. *p < 0.05, **p < 0.01, ***p < 0.001, ****p < 0.0001.

**Fig. 4:**
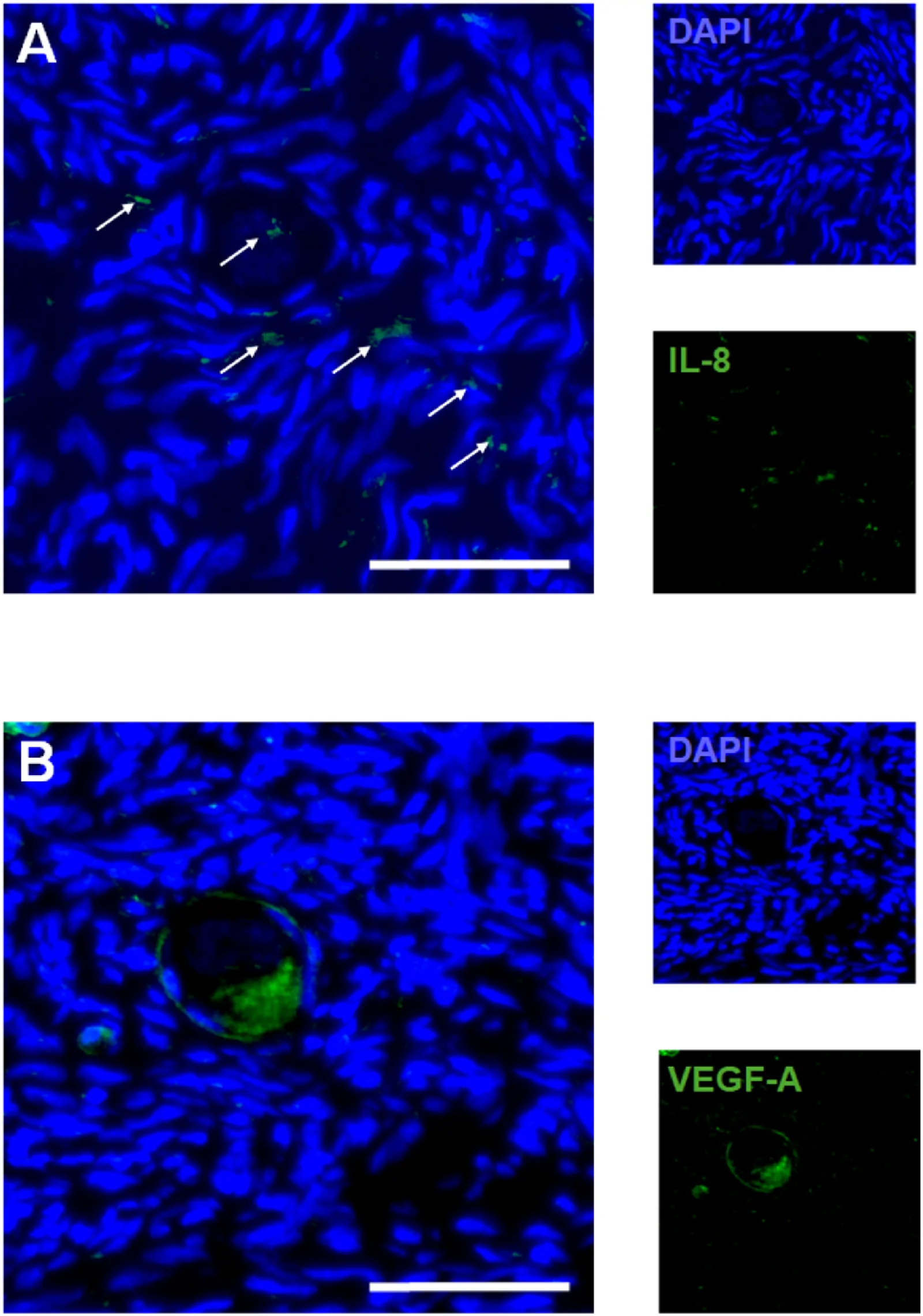
**Representative images of IL-8 and VEGF-A stained primordial follicles.** Ovarian cortical tissue was stained for IL-8 or (A) VEGF-A (B) and nuclear counterstaining with DAPI. While IL-8 staining is rather delicate (white arrows mark IL-8 staining), VEGF-A shows stronger staining patterns. In order to enhance the signal-to-background ratio, the blocking reagent TrueBlack was used. The respective staining controls were used to adjust the display settings. Images show primordial follicles. The white bar marks a scale of 50 μm.

As a logic consequence two complete IHF stainings were conducted in order to assess the quality. VEGF-A staining serving stronger signals and IL-8 staining with lower overall signal were chosen as staining examples. Compared to when no autofluorescence quencher was applied, TrueBlack was able to significantly increase the SBR (signal-to-background ratio) of the IL-8 staining analyzing whole tissue (*p < 0.05, see Fig. 3B) by reducing the background signal significantly (*p < 0.05, see Fig. 3B). Similarly, the SBR of certain structures, such as follicles (see Fig. 4A) and vessels was increased by TrueBlack, caused by a significant decrease of the background (****p < 0.0001 and **p < 0.01, respectively, see Fig. 3C). No significant change of the IL-8 signal was found.

The overall stronger staining of VEGF-A also profited by the autofluorescence blockage. A slight increase of the SBR in the whole tissue was caused by a significant drop of the background (***p < 0.001, see Fig. 3D). Similarly, certain structures, such as follicles, showed improved SBR due to significantly decreased background (***p < 0.001, see Figs. 3E and 4B). In strongly stained structures such as vessels the staining excelled the background anyways so that barely any effect was seen in the SBR. Nonetheless, the reduction of the background here as well as in Fig. 2C alleviates the separation of background and signal in any case, since no significant impact on the signal intensity was found.

## 4 Discussion

### 4.1 Autofluorescence in ovaries

In order to provide conclusive IHF images and analyses, the blocking of autofluorescence enhances the significance and avoids false-positive signals, which can possibly lead to imprecise conclusions. Due to the complexity of the ovarian biology, cell culture models barely allow its study. Thus, the assessment of parameters in *ex vivo* tissue allows important insights at least a snapshot of ongoing processes. Examining tissue represents the 2D/3D-arranged structures and cell types in their actual position. This allows to observe structural distribution of cells or factors and their association to other structures.

Applying our IHF protocol concerning fixation, demasking and staining, all four kits showed a significant reduction of the general autofluorescence signal. The strongest reduction was achieved by the application of the TrueBlack kit. Most importantly, the prominent autofluorescence signals of blood vessels and follicles signals were reduced sufficiently.

Different structures excite autofluorescence including matrix components and lipofuscin. These are especially found in the ovarian cortex in general as well as in blood vessels and follicles [12]. Ovarian cortical tissue is relatively dense as it contains high amounts of matrix components. Although it varies throughout the cycle, concentric layers of several collagen subtypes create a matrix-rich environment [13]. A general occurrence of autofluorescence was seen accordingly.

Certain structures showed even more autofluorescence signal, such as blood vessels and follicles. The wall of blood vessels is enriched in matrix components. In general, vascular basement membrane consists of laminin, nidogen and heparan sulfate proteoglycans [14]. In the human ovary, collagen IV is enriched around blood vessels [15]. Likewise, follicles are embedded in a basal membrane built of matrix components. The follicular basal membrane surrounds the granulosa cells. As the follicles grow throughout folliculogenesis, the basal membrane thickens and changes [16,17]. Moreover, strong autofluorescent signals were observed in the oocytes.

Lipofuscin is found in diverse structures of the ovary. It is a product of lipid metabolism and is found in oocytes, artretic follicles and especially in the *corpus luteum* [18]. Moreover, in mouse ovaries, phagocytes show increased autofluorescence as their phagosomes contain lipofuscin [19,20]. As lipofuscin is indigestible, it progressively accumulates especially in ageing cells. In mice, it was shown to be accumulated in the ovaries with rising age [21].

### 4.2 Autofluorescence blockage

There are several chemicals which can reduce autofluorescence: Copper Sulfate Solution, Sodium Borohydride, Sudan Black B, Ammonia and Trypan Blue.

Sudan Black B has been reported to bind lipofuscin and thereby mask its autofluorescence [21]. Since lipofuscin is formed of oxidized proteins and lipids, the slightly basic Sudan Black B reacts with it. Since lipofuscin consists of oxidized proteins and lipids, Sudan Black B is thought to interact with these components. According to the manufacturers, TrueBlack and MaxBlock are based on a similar principle. Therefore, the superior autofluorescence reduction observed in our study may, at least in part, be explained by masking of lipofuscin. However, the present study was not designed to investigate the underlying mechanisms of action, and these explanations remain speculative.

Based on the manufacturers' information and the characteristics of the reagents, ReadyProbes and TrueVIEW appear to be based on copper sulfate solutions. Copper ions have been reported to reduce aldehyde-induced autofluorescence by forming complexes with fluorescent Schiff bases [22]. The comparatively weaker performance observed in our study may therefore reflect differences in the predominant sources of autofluorescence in human ovarian tissue. However, this was not investigated experimentally.

Although the observed differences between the reagents are consistent with mechanisms proposed in previous studies and by the manufacturers, the present study was designed to evaluate their performance rather than their mechanisms of action. Therefore, the mechanistic explanations discussed above should be regarded as possible interpretations of our findings.

### 4.3 Sucrose

The incubation in sucrose dehydrates the tissue in order to prevent the tissue from freezing damage. Sucrose solutions with a concentration of 10 % and above exerts an osmotic pressure. Here, as subsequent incubation in 15 % sucrose and 30 % sucrose solution was performed. Surprisingly it mostly led also to a reduction of the autofluorescent signal. In case of samples treated with TrueBlack, incubation in sucrose led to an autofluorescence decrease in all channels.

In general, dehydration of the tissue also affects the quality of microscopic imaging. Tissue consists of a variety of structures on cellular, extracellular and intracellular level. Since all these structures show different refractive indices, it causes scattering of light. Optical clearance is increased, when refractive indices throughout are matching [23]. Moreover, saccharides, such as sucrose interact with collagen fibers and help them to dissociate. This as well reduces light scattering. However, both mechanisms are reversible and may not explain the additional reduction of autofluorescent signal.

Nonetheless, under the experimental conditions applied in this study, incubation in sucrose solution was associated with a reduction in autofluorescence. Therefore, sucrose incubation may provide an additional benefit beyond cryoprotection when preparing human ovarian cryosections.

### 4.4 VEGF-A and IL-8 in ovarian tissue

Strong staining, such as VEGF-A staining in ovarian tissue, as well as weak staining such as IL-8 staining in ovarian tissue benefit from reduced autofluorescence. As it lowers the background, the SBR is increased and the staining can stand out more strongly. Especially locating weak staining signals may be disturbed or even impossible in the presence of high autofluorescence signals as seen in follicles and vessels.

The expression of VEGF-A in ovarian tissue was described before [6,7,24]. As the growth factor can bind to matrix components, which are highly abundant in the ovary, the signal is known and expected to be relatively strong. Vessels, follicles as well as various cells show a strong VEGF-A staining.

In contrast, IL-8 staining showed a generally weaker staining. The cytokine IL-8, also known as chemokine CXCL8, is enriched in the follicular fluid [25], outermost ovarian capsule, in theca cells and blood vessels of the theca layer [26,27]. Low IL-8 expression is found in other cells such as granulosa or stromal cells [27].

### 4.5 Limitations

A limitation of this study is the limited number of biological replicates (tissue samples from different patients). Inter-individual variability arising from factors such as age, disease status, tissue quality and integrity, and fixation conditions can influence autofluorescence characteristics. While overall autofluorescence was assessed in samples from 4 patients, the detailed evaluation of blood vessels, follicles, and staining patterns was performed using tissue from a single patient. Therefore, these findings should be interpreted with caution and validated in a larger cohort.

A limitation of the present study is that the autofluorescence quenching reagents were evaluated as complete commercial products under the manufacturers' recommended protocols. Consequently, the study was not designed to investigate the contribution of individual protocol parameters, such as reagent composition, concentration, incubation time, or temperature, to the observed quenching performance. Since each blocking reagents react with other autofluorescent structures, a combination of several blocking reagents may have led to even better blocking of autofluorescence. This was not tested throughout this project.

Another limitation of the present study is that the performance of the evaluated reagents was assessed based on the overall reduction of tissue autofluorescence rather than their efficacy against specific endogenous fluorophores, such as lipofuscin, collagen, or erythrocytes. As multiple endogenous fluorophores contribute simultaneously to ovarian tissue autofluorescence, distinguishing the contribution of individual fluorophores was beyond the scope of this study. Future studies combining histological characterization with spectral imaging may provide further insight into reagent-specific quenching of different autofluorescent tissue components.

Furthermore, all image acquisition was performed using a single microscope platform with standardized imaging settings. Although this ensured direct comparability between the evaluated reagents, the performance of autofluorescence quenching strategies may vary depending on the imaging system, optical configuration, and acquisition parameters. Therefore, validation on additional microscope platforms would further strengthen the generalizability of the present findings.

For cryosections, it is not necessary to fix the tissue. Whereas fixation with formaldehyde is rather used for paraffin sections. We chose this protocol as we chose to make sure to have a strong conservation of the antigens and the morphology.

The workflow applied in this study, including standardized tissue processing, fixation, autofluorescence quenching, image acquisition, and analysis, may provide a practical starting point for immunofluorescence studies using scarce human ovarian tissue. Future studies may further optimize individual protocol steps depending on the experimental question and staining panel.

## 5 Conclusion

All used blocking reagents reduced autofluorescence. In this study, we systematically compared four commercially available reagents to reduce autofluorescence in our specific experimental conditions. In our case, examining human ovarian tissue with the applied fixation, antigen retrieval and microscope settings, TrueBlack, which is based on Sudan Black B, provided the best results. Nonetheless other autofluorescence reducing kits, such as TrueVIEW and Ready Probes showed comparable results.

Given the high demand for research on human ovarian tissue and the exceptional rarity of samples, methodological experiences should be shared.

## CRediT authorship contribution statement

**Rebekka Einenkel-Ulrich:** Conceptualization, Data curation, Formal analysis, Investigation, Methodology, Project administration, Supervision, Visualization, Writing – original draft, Writing – review & editing. **Johanna Schlingheider:** Data curation, Formal analysis, Investigation, Methodology, Visualization, Writing – original draft, Writing – review & editing. **Cara Maria Färber:** Data curation, Resources, Writing – review & editing. **Andreas Schallmoser:** Data curation, Resources, Writing – review & editing. **Nicole Sänger:** Funding acquisition, Resources, Writing – review & editing.

## Ethics declaration

Written informed consent to take part in the study and to publish the article has been obtained from all participants or their legal representatives. The privacy rights of participants have been observed.

This study included organ or tissue donors. This study includes human biological material and consent was obtained by donors, or their next of kin or legal representatives, for use in this study and for publication of the article. The samples used in this research were not sourced from executed prisoners or prisoners of conscience.

This study was performed in compliance with relevant laws, regulatory frameworks and guidelines where the research took place. This study was approved by the Ethics Committee of the Medical Faculty of the University Hospital Bonn, University Bonn. (Approval No. 007/09)

## Funding

This research did not receive any specific grant from funding agencies in the public, commercial, or not-for-profit sectors. This study was funded by intramural funding of the University Hospital Bonn.

## Declaration of competing interests

The authors declare that they have no known competing financial interests or personal relationships that could have appeared to influence the work reported in this paper.

## Data Availability

Data will be made available on request.
